# Nano-Se exhibits limited protective effect against heat stress induced poor breast muscle meat quality of broilers compared with other selenium sources

**DOI:** 10.1186/s40104-024-01051-2

**Published:** 2024-07-08

**Authors:** Jinzhong Jing, Jiayi Wang, Qian Wu, Shenggang Yin, Zhen He, Jiayong Tang, Gang Jia, Guangmang Liu, Xiaoling Chen, Gang Tian, Jingyi Cai, Bo Kang, Lianqiang Che, Hua Zhao

**Affiliations:** 1 Key Laboratory for Animal Disease-Resistance Nutrition of Ministry of Education, of China Ministry of Agriculture and Rural Affairs, of Sichuan Province, Animal Nutrition Institute, Sichuan Agricultural University, Chengdu, 611130 Sichuan China; 2 College of Animal Science and Technology, Sichuan Agricultural University, Chengdu, 611130 Sichuan China

**Keywords:** Broilers, Heat stress, Meat quality, Mitochondrial stress, Nano-Se, Se sources

## Abstract

**Background:**

At present, heat stress (HS) has become a key factor that impairs broiler breeding industry, which causes growth restriction and poor meat quality of broilers. Selenium (Se) is an excellent antioxidant and plays a unique role in meat quality improvement. Recent years, nano-selenium (NanoSe) has received tremendous attention in livestock production, due to its characteristic and good antibacterial performance in vitro. Here, we developed the heat stressed-broiler model to investigate the protective effects of NanoSe on growth performance and meat quality of broilers and compare whether there are differences with that of other Se sources (Sodium selenite, SS; Selenoyeast, SeY; Selenomethionine, SeMet).

**Results:**

HS jeopardized the growth performance and caused poor meat quality of breast muscle in broilers, which were accompanied by lowered antioxidant capacity, increased glycolysis, increased anaerobic metabolism of pyruvate, mitochondrial stress and abnormal mitochondrial tricarboxylic acid (TCA) cycle. All Se sources supplementation exhibited protective effects, which increased the Se concentration and promoted the expression of selenoproteins, improved the mitochondrial homeostasis and the antioxidant capacity, and promoted the TCA cycle and the aerobic metabolism of pyruvate, thus improved the breast muscle meat quality of broilers exposed to HS. However, unlike the other three Se sources, the protective effect of NanoSe on meat quality of heat stressed-broilers was not ideal, which exhibited limited impact on the pH value, drip loss and cooking loss of the breast muscle. Compared with the other Se sources, broilers received NanoSe showed the lowest levels of slow MyHC, the highest levels of fast MyHC and glycogen, the highest mRNA levels of glycolysis-related genes (*PFKM* and *PKM*), the highest protein expression of HSP60 and CLPP, and the lowest enzyme activities of GSH-Px, citroyl synthetase (CS) and isocitrate dehydrogenase (ICD) in breast muscle. Consistent with the SS, the Se deposition in breast muscle of broilers received NanoSe was lower than that of broilers received SeY or SeMet. Besides, the regulatory efficiency of NanoSe on the expression of key selenoproteins (such as SELENOS) in breast muscle of heat stressed-broilers was also worse than that of other Se sources.

**Conclusion:**

Through comparing the meat quality, Se deposition, muscle fiber type conversion, glycolysis, mitochondrial homeostasis, and mitochondrial TCA cycle-related indicators of breast muscle in heat stressed broilers, we found that the protective effects of organic Se (SeY and SeMet) are better than that of inorganic Se (SS) and NanoSe. As a new Se source, though NanoSe showed some protective effect on breast muscle meat quality of heat stressed broilers, the protective effect of NanoSe is not ideal, compared with other Se sources.

**Supplementary Information:**

The online version contains supplementary material available at 10.1186/s40104-024-01051-2.

## Background

Poultry meat is a key protein source for human. In 2021, poultry meat accounts for more than 38% of the global meat production, and broiler chicken is the main source of poultry meat [1]. However, recent years, the global mean surface air temperature keeps rising, and extreme heat events occur frequently [2]. Therefore, the high-density intensive poultry industry faces enormous challenge of heat stress (HS). Evidences suggest that HS compromises the growth performance, and impairs meat quality of broilers [3–5]. HS caused poor meat quality of broilers mainly due to the mitochondrial stress, which triggers excessive reactive oxygen species (ROS) production and causes oxidative stress, thus lowers the antioxidant capacity and reduces the pH of meat [6–8]. Excessive ROS changes the protein conformation and promotes the formation of polymerized or cross-linked proteins, thus affects the tenderness and water-holding capacity of meat [9]. Besides, previous study shows that HS induced mitochondrial stress is accompanied by the abnormal mitochondrial tricarboxylic acid (TCA) cycle, which suppress the aerobic metabolism of pyruvate in liver of broilers [10]. HS also affects the proportion of muscle fibers and promotes the anaerobic metabolism of pyruvate, thus induces the accumulation of lactic acid in muscle and lowers the pH of meat samples [11]. Hence, it is reasonable to expect that alleviation of mitochondrial stress may be an effective way to enhance the antioxidant capacity, promote the aerobic metabolism of pyruvate, and improve the meat quality of broilers exposed to HS.

Selenium (Se) is an excellent antioxidant and plays a unique role in HS resistance and meat quality improvement. Se participates in the formation of antioxidant system of animals and contributes to the maintenance of mitochondrial homeostasis in skeletal muscle [12, 13]. Se deficiency induces breast muscle injury and redox imbalance in broilers exposed to HS [14]. Se performs its anti-stress effect mainly through promoting the expression of selenoproteins, such as the glutathione peroxidase family (GPXs) [10, 15, 16]. The increased expression of selenoproteins that involved in the regulation of redox homeostasis contribute to clearing excessive ROS and recovering mitochondrial homeostasis. Generally, the expression of selenoproteins in skeletal muscle of animals is effectively regulated by dietary Se supplementation [17]. At present, the sodium selenite (SS), selenoyeast (SeY) and selenomethionine (SeMet) are commonly used as Se sources in livestock production, and the deposition efficiencies of these Se sources in skeletal muscle of broilers have been verified [18]. Besides, the pathway that selenoproteins synthesized by these Se sources in animals is clear [19]. Recent years, nano selenium (NanoSe) has received tremendous attention in livestock production, due to its characteristic and good antibacterial performance in vitro. For poultry nutrition, studies show that NanoSe can increase Se concentration and improve the antioxidant capacity in skeletal muscle of broilers [20, 21]. However, the question of how NanoSe is converted into active selenoproteins in animals is not resolved [22]. Compared with other Se sources (SS, SeY, or SeMet), the selenoproteins conversion efficiency of NanoSe needs to be verified, and whether NanoSe can effectively protect meat quality of broilers exposed to HS remain unclear. Therefore, the heat stressed-broiler model was developed to investigate: 1) whether NanoSe can effectively improve breast muscle meat quality of heat stressed broilers; 2) whether NanoSe can effectively relieve mitochondrial stress and improve glycolysis in breast muscle of heat stressed broilers; and 3) the selenoprotein conversion efficiency and protective effect of NanoSe, compared with other Se sources.

## Materials and methods

### Animal, diet and experimental design

The trial contained two stages, pre-feeding period (1–21 d old) and test period (22–42 d old). In pre-feeding period, 600 Arbor Acres male broilers (1 d old, 45 ± 5 g) were divided into 6 groups with 10 replicates of 10 broilers per replicate. Broilers in 2 groups were feed on basal diet without additional Se supplementation, broilers in other 4 groups were feed on basal diet supplied with 0.3 mg Se/kg in form of SS (Se concentration: 0.45%), SeY (Se concentration: 2000 mg/kg), SeMet (Se concentration: 2000 mg/kg) or NanoSe (Se concentration: 3,000 mg/kg, particle size: 60 nm), respectively. All broilers were housed in the same temperature and relative humidity, the temperature was gradually decreased from 33 to 22 °C (from 1 to 21 d), the relative humidity was kept at 50%.

After 21 d, 80 broilers (650 ± 50 g) in each group were selected for the further test (*n* = 8 for each group). Broilers in the control group (CON) were hold on the thermoneutral environment (Temperature: 22 ± 2 ℃; Relative humidity: 50% ± 5%) and further feed on basal diet. Broilers in other 5 group were raised in the hyperthermal environment (Temperature: 33 ± 2 ℃; Relative humidity: 65% ± 5%) and further feed on basal diet (HS) or basal diet supplied with 0.3 mg Se/kg SS (HS + SS), SeY (HS + SeY), SeMet (HS + SeMet) or NanoSe (HS + NanoSe). The SS, SeY, SeMet and NanoSe used in the present study were obtained from Chengdu Shuxing Feedstuff Co., Ltd. (Sichuan, China), Angel Yeast Co., Ltd. (Sichuan, China), Sichuan Sinyiml Biotechnology Co., Ltd. (Sichuan, China) and Sichuan Chelota Biotech Co., Ltd. (Sichuan, China), respectively. The trial was finished at 42 d. The basal diets in each stage were formulated according to the Nutrient Requirements of Poultry (NRC, 1994) [23], the composition and nutrient levels of basal diets were shown in Table 1, and the Se concentration in diets were determined and shown in Additional file 1: Fig. S1.

**Table 1:** Composition and nutrient levels of the basal diet (air-dry basis)

Ingredients, %	1–21 d	22–42 d
Corn (CP 8%)	58.50	63.14
Soybean meal (CP 44.2%)	33.42	31.20
Fish meal (CP 62.5%)	1.80	-
Soybean oil	2.35	2.00
CaCO_3_	1.40	1.25
CaHPO_4_	1.45	1.58
NaCl	0.40	0.35
Lys-HCl	0.04	0.02
DL-Met	0.26	0.16
L-Thr	0.03	-
Choline chloride	0.15	0.10
Premix^1^	0.20	-
Premix^2^	-	0.20
Total	100.00	100.00
Nutrient composition, %		
Metabolic energy, MJ/kg	12.27	12.33
Crude protein	20.89	19.01
Total Ca	1.01	0.90
Total P	0.68	0.65
Available P	0.41	0.38
Lys	1.15	1.00
Met	0.58	0.45
Met + Cys	0.91	0.76
Thr	0.82	0.72
Val	0.96	0.87
Trp	0.24	0.22

^1^Premix for 1–21 d broiler provided (per kg): Cu (CuSO_4_·5H_2_O), 8 mg; Fe (FeSO_4_·7H_2_O), 100 mg; Mn (MnSO_4_·H_2_O), 120 mg; Zn (ZnSO_4_·H_2_O), 100 mg; I (KI), 0.7 mg; Vitamin A, 8,000 IU; Vitamin D_3_, 2000 IU; Vitamin E, 20 IU; Vitamin K_3_, 3.2 mg; Vitamin B_1_, 2 mg; Vitamin B_2_, 6.4 mg; Vitamin B_6_, 4 mg; Vitamin B_12_, 0.2 mg; D-Biotin, 1.1 mg; D-Pantothenic acid, 12 mg; Folic acid, 1 mg; Nicotinamide, 40 mg

^2^Premix for 22–42 d broiler provided (per kg): Cu (CuSO_4_·5H_2_O), 8 mg; Fe (FeSO_4_·7H_2_O), 80 mg; Mn (MnSO_4_·H_2_O), 100 mg; Zn (ZnSO_4_·H_2_O), 80 mg; I (KI), 0.7 mg; Vitamin A, 6,000 IU; Vitamin D_3_, 1,500 IU; Vitamin E, 15 IU; Vitamin K_3_, 2.4 mg; Vitamin B_1_, 1.5 mg; Vitamin B_2_, 4.8 mg; Vitamin B_6_, 3 mg; Vitamin B_12_, 0.15 mg; D-Biotin, 0.825 mg; D-Pantothenic acid, 9 mg; Folic acid, 0.75 mg; Nicotinamide, 30 mg

Broilers in each replicate of different groups were separated housed in cages with the same size of length × width × height, 100 cm × 80 cm × 60 cm. The temperature and relative humidity in this study were controlled using the poultry farm monitoring system (ROTEM AC-2000 PLUS, AgroLogic Ltd., Israel). The electric hot air blower (50 kW, Shandong Machinery Co., Ltd., Qingzhou, China) was used to provide the extra thermal energy. The fog machine (PC-2804, Taizhou Paichi Machinery Co., Ltd., Taizhou, China) was used to provide the extra relative humidity. During the trial, all broilers were free access to diet and water.

### Respiration rate

The respiration rate of broilers was tested every 3 d in the test period by using a manual mechanical counter. One broiler in each replicate of different groups were selected (a total of 48 broilers), tracked and recorded for 30 s. Then, the respiration rate of broilers in each group was quantified and expressed as breaths/min.

### Growth performance

The total feed intake and final body weight (BW) of broilers in each replicate were determined at the last of the trial. Then the average daily feed intake (ADFI), average daily body gain (ADG) and feed intake/body gain (F/G) were calculated as followed: ADFI = total feed intake of each replicate (g)/test days/total number of broilers in each replicate; ADG = total body gain of each replicate (g)/test days/total number of broilers in each replicate; F/G = ADFI/ADG.

### Diet sample collection

According to the experimental design, diets were formulated for two times, diet samples (200 g) from each treatment group were collected in the Ziplock bag and stored at −20 °C for Se concentration analysis.

### Breast muscle rate calculation and sample collection

After 42 d, 6 broilers in each treatment group (the body weight of each broiler was closed to the average body weight of each treatment group) were selected and fasted overnight. These broilers were weighed and recorded as live weight, then slaughtered. The breast muscle of each broiler was completely detached, weighed and recorded as breast muscle weight. The breast muscle rate was calculated: Breast muscle rate (%) = breast muscle weight/live weight × 100. Little part of the right breast muscle of each broiler was collected in the sterile tube, and stored at −80 °C for laboratory analysis.

### Meat quality of breast muscle

The meat quality indicators in the present study contains the pH value, meat color, drip loss, cooking loss and peak shear force, and the measurement methods were referred to a previous study [24]. The detailed descriptions are as follows.

#### pH and meat color

A part of the left breast muscle of each selected broiler was cut and used to measure the pH value and meat color by using the portable pH meter (pH-Star, Matthäus, Pöttmes, Germany) and the Minolta Chromameter CR-300 (Minolta Camera, Osaka, Japan). Meat color includes lightness (L*), redness/greenness (a*) and yellowness/blueness (b*). Breast muscle samples were cut and placed for 45 min, the pH and meat color were measured in three different points. Then, these meat samples were subsequently placed in Ziplock bags and stored at 4 °C, after 24 h, the pH and meat color were measured again. The calculated mean values of three different points in each meat sample were used for statistical analysis.

#### Cooking loss

Briefly, a small piece of the left breast muscle (about 30 g) from each selected broiler were cut and stored at 4 °C. After 24 h, these breast muscle samples were weighed and recorded as *W*_*a*_. Then, these samples were independently placed in Ziplock bags and cooked in a 90 °C water bath. The kerosene thermometer was used to monitor the internal temperature of each sample. When the internal temperature reached 70 °C, took out these meat samples. After naturally cooled to room temperature, the water in each sample surface was absorbed by using the filter paper. Then, these meat samples were weighed and recorded as *W*_*b*_. The cooking loss of each sample was calculated: Cooking loss (%) = (*W*_*a*_ − *W*_*b*_)/*W*_*a*_ × 100.

#### Drip loss

After the breast muscle was weighed, a fresh thick slice (5 cm × 3 cm × 0.5 cm) was cut from the right breast muscle, weighed and recorded as *W*_1_. Then, these thick slices were independently placed in Ziplock bags and suspended in a 4 °C freezer. After 24 and 48 h, the water in each sample surface was absorbed by using the filter paper, then, these samples were reweighed and recorded as *W*_2_ and *W*_3_. The drip loss of each sample was calculated: 24 h drip loss (%) = (*W*_1_ − *W*_2_)/*W*_1_ × 100; 48 h drip loss (%) = (*W*_1_ − *W*_3_)/*W*_1_ × 100.

#### Peak shear force

After measured the 24 h meat color and pH, the left breast muscle samples were used to determine the peak shear force. All samples were independently placed in Ziplock bags and cooked in a 90 °C water bath. The kerosene thermometer was used to monitor the internal temperature of each sample. When the internal temperature reached 70 °C, took out these meat samples. After naturally cooled to room temperature, these samples were stored at 4 °C. After 24 h, 5 cores (1.2 cm diameter) from each sample were collected using the same sampler. Then, these cores were sheared by using the Texture Analyzer (XT2, Stable Micro Systems Ltd., Godalming, Surrey, UK). The peak shear force of each core was recorded, and the mean values of the 5 cores from the same breast muscle sample were used for statistical analysis.

### Selenium concentration in diets and breast muscle

The Se concentration in diet samples and breast muscle samples were determined by using the hybrid generation-atomic fluorescence spectrometer (AFS-230E, Beijing Haiguang instrument, China). The Se concentration in diet samples and breast muscle samples were measured according to the national standard of China (GB/T 13883–2008, GB 5009.93–2010) [25, 26]. The sample pretreatments were referred to a previous study [27].

### Biochemical analysis of the breast muscle

The concentration of glycogen, glucose, lactic acid, pyruvate, mitochondrial citric acid (CA), malondialdehyde (MDA) and adenosine triphosphate (ATP) in breast muscle samples were determined by using the corresponding assay kits (No. A043-1-1, A154-1-1, A019-2-1, A081-1-1, A128-1-1, A003-1, A095-1-1, Nanjing Jiancheng Bioengineering Institute, Nanjing, China). The concentration of acetyl-CoA and oxaloacetate (OA), and the enzyme activity of citroyl synthetase (CS) and isocitrate dehydrogenase (ICD) in breast muscle samples were measured by using the enzyme-linked immunosorbent assay (ELISA) kits (No. YJ711921, YJ792302, YJ292912, YJ719222, Shanghai Enzyme-linked Biotechnology Co., Ltd., Shanghai, China). The concentration of ROS in breast muscle samples were determined by using an ELISA kit (No. MM-6012001, Jiangsu Meimian Industrial Co., Ltd., Jiangsu, China). The enzyme activity of lactic dehydrogenase (LDH), succinodehydrogenase (SDH), malic dehydrogenase (MDH), glutathione peroxidase (GSH-Px), total antioxidant capacity (T-AOC) and total superoxide dismutase (T-SOD) in breast muscle samples were measured by using the corresponding assay kits (No. A020-1-2, A022-1-1, A021-2-1, A005, A015-1, A001-1-1, Nanjing Jiancheng Bioengineering Institute, Nanjing, China). The protein concentration of sample extraction solution was measured by using a commercial assay kit (No. A045-3, Nanjing Jiancheng Bioengineering Institute, Nanjing, China). The sample pretreatments were referred to these kit instructions.

### RT-PCR analysis of mRNA expression in breast muscle

The mRNA expressions of the target genes were determined by using the RT-PCR analysis. The sample preparation method and PCR procedures were referred to the previous study [13]. Briefly, the total RNA in each sample was extracted by using the RNAiso Plus (No. 9109, Takara, Dalian, China). Nucleic acid electrophoresis was used to ensure the RNA quality. Then, a total of 1,000 ng RNA was used for cDNA synthesis by using the ExonScript RT SuperMix kit (No. A502-02, EXONGEN, Chengdu, China). The RT-PCR was performed in QuantStudio 5 Flex system (Applied Biosystems, CA, USA), and the final reaction volume was 10 μL by using the Fast SYBR Green qPCR Master Mix UDG kit (No. A402-1, EXONGEN, Chengdu, China). *β-Actin* was selected as the reference gene to calculate the relative mRNA expression of these target genes. All of the primers for target genes and *β-Actin* were picked from the National Center for Biotechnology Information (https://www.ncbi.nlm.nih.gov/), and listed in Additional file 2: Table S1.

### Western blot analysis of protein expression in breast muscle

Sample preparation method and western blot process were referred to the previous study [15]. Briefly, the total protein of each sample was extracted by using the radio immunoprecipitation assay (RIPA) buffer with phenylmethanesulfonyl fluoride (PMSF) protease inhibitor buffer (Beyotime, Shanghai, China). Then, the total protein concentration of each sample was adjusted to 6 μg/μL. 400 μL of the above samples were denatured in the water bath (95 °C) for 10 min after mixed with 100 μL SDS-PAGE sample loading buffer (5 ×) (No. P0015L, Beyotime, Shanghai, China). The 30 μg protein from each sample was used for the western blot process. The primary antibodies were shown in Additional file 2: Table S2, and the secondary antibodies were anti-rabbit or anti-mouse IgG (dilution ratio: 1:5,000, Proteintech Group, IL, USA). The bands were visualized by using an enhanced chemiluminescence system (Bio-Rad, CA, USA), and analyzed using the Image Lab™ software (Bio-Rad, CA, USA).

### Statistical analysis

The SPSS 27.0 (SPSS Inc., Chicago, USA) was used to perform the statistical analysis. Results are expressed as means with standard error of the mean (SEM) or standard deviation (SD). The independent sample *t*-test was used to distinguish whether there is a difference between CON and HS group. And the one-way ANOVA followed by Tukey’s multiple range test was used to compare whether there are differences among these HS groups (HS, HS + SS, HS + SeY, HS + SeMet, HS + NanoSe). If the *P*-values of *t*-test or ANOVA less than 0.05 were considered statistically significant. Principal component analysis of the selenotranscriptome was also accomplished by SPSS 27.0 (SPSS, Inc., Chicago, USA). The correlation analysis was performed by using the Origin 2021 (OriginLab, Northampton, MA, USA). Except for the growth performance and heat map of correlation analysis, results were plotted by using the GraphPad Prism Version 8 software (Graphpad software, LLC, San Diego, USA).

## Results

### Respiration rate and growth performance

**Fig. 1:**
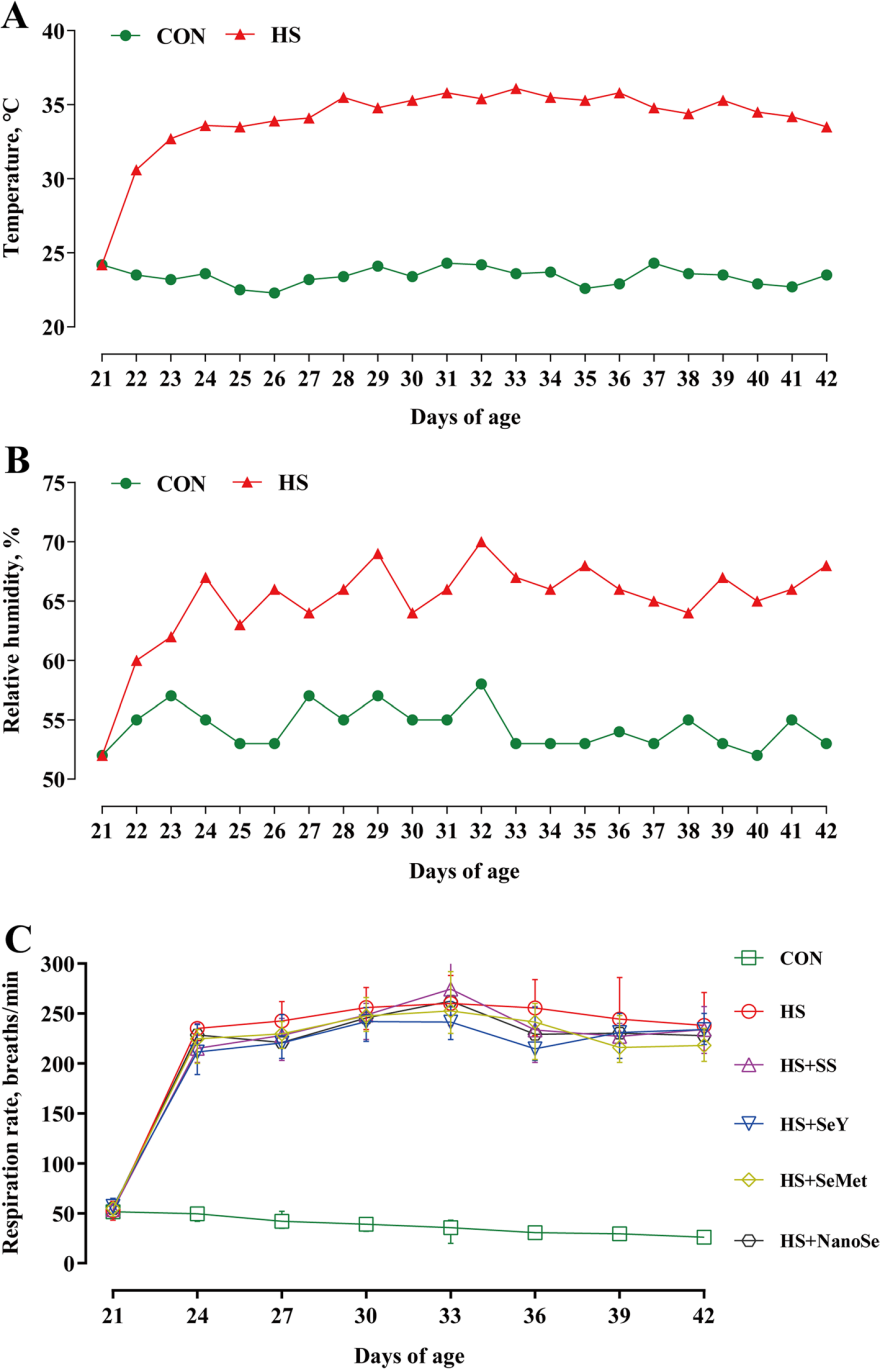
Room ambient temperature (**A**), room relative humidity (**B**), and respiration rate of broilers (**C**). Results for respiration rate was expressed as means with SD (*n* = 8)

We measured the respiration rate and growth performance of broilers to assess the influence of HS. In the present study, HS increased the respiration rate (Fig. 1C) and caused poor growth performance of broilers (Table 2). Broilers exposed to HS showed the lower (*P* < 0.05) final BW, ADFI and ADG, while the higher (*P* < 0.05) F/G after treated for 21 d (Table 2). Consistent with the other three Se sources, NanoSe exhibited no impact (*P* > 0.05) on the respiration rate, as well as the growth performance of broilers exposed to HS.

**Table 2:** Growth performance of broilers

Item	Groups						SEM	*P*-value	
	CON	HS	HS + SS	HS + SeY	HS + SeMet	HS + NanoSe		*P*^1^	*P*^2^
Pre-feeding period (1–21 d)									
BW, g/bird									
1 d	46.75	46.80	46.85	47.00	46.60	46.80	0.06	0.833	0.353
21 d	652.16	659.35	664.45	647.22	653.05	647.74	4.97	0.588	0.204
ADFI, g/d	44.35	44.36	44.75	43.58	43.68	42.96	0.40	0.996	0.115
ADG, g/d	28.83	29.17	29.41	28.58	28.88	28.62	0.21	0.776	0.537
F/G	1.54	1.52	1.52	1.52	1.51	1.50	0.01	0.825	0.885
Test period (22–42 d)									
BW, g/bird									
22 d	658.50	656.94	655.06	654.69	657.44	653.50	0.66	0.612	0.307
42 d	2,229.29^*^	1,649.71	1,643.00	1,669.99	1,625.93	1,654.71	7.56	< 0.001	0.474
ADFI, g/d	130.49^*^	97.78	96.33	96.20	96.40	97.31	0.48	< 0.001	0.831
ADG, g/d	74.80^*^	47.27	47.04	48.35	46.12	47.68	0.36	< 0.001	0.399
F/G	1.74^*^	2.07	2.05	1.99	2.09	2.04	0.02	< 0.001	0.333

Results for BW, ADFI, ADG and F/G were expressed as mean with SEM (for 1–21 d, *n* = 10; for 22–42 d, *n* = 8). *P*^1^ was the *t*-test *P*-value between CON and HS group. *P*^2^ was the ANOVA *P*-value among these HS groups

*BW* Body weight, *ADFI* Average daily feed intake, *ADG* Average daily body gain, *F/G* Feed intake/body gain

^*^ There was a significantly difference between CON and HS groups (*P* < 0.05)

### Breast muscle rate, Se concentration and meat quality

**Fig. 2:**
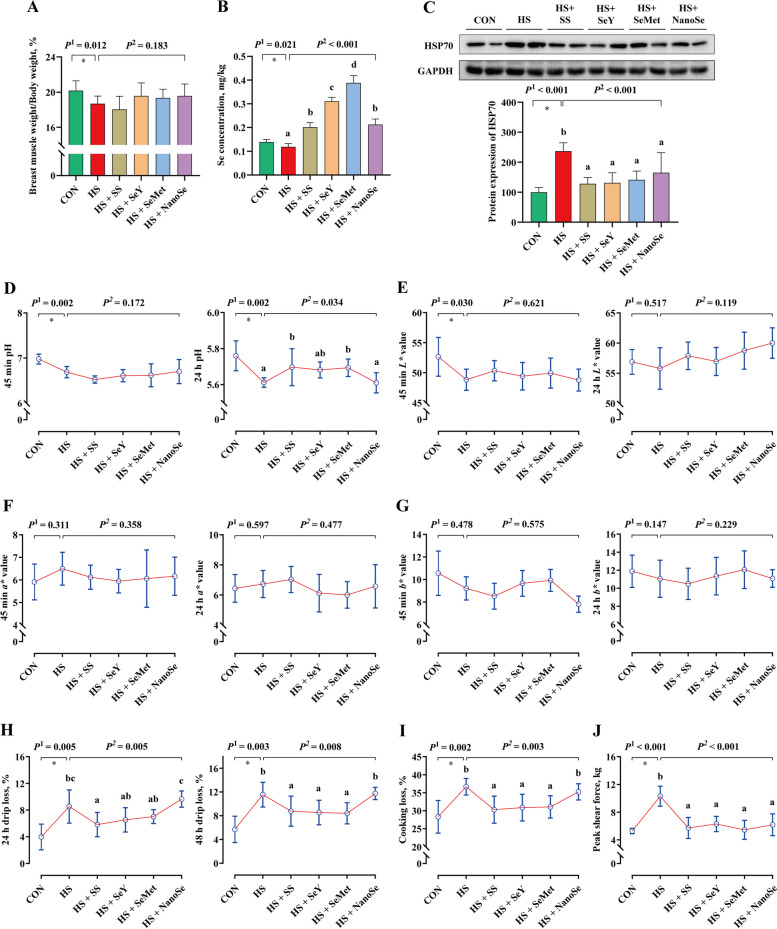
Breast muscle weight and meat quality of broilers. **A** Breast muscle rate of broilers; **B** Se concentration in breast muscle; **C** Protein expression of HSP70; **D** pH value of breast muscle; **E** L* value of breast muscle; **F** a* value of breast muscle; **G** b* value of breast muscle; **H** Drip loss of breast muscle; **I** Cooking loss of breast muscle; **J** Peak shear force of breast muscle. Results were expressed as means with SD (*n* = 6). *P*^1^ was the *t*-test *P*-value between CON and HS group. *P*^2^ was the ANOVA *P*-value among these HS groups. * means there was a significantly difference between CON and HS group (*P* < 0.05). Different letters indicate significant difference between each HS group (*P* < 0.05)

The breast muscle is the main component of skeletal muscle in broilers, which is the main edible part of broilers. In the present study, HS decreased (*P* < 0.05) the breast muscle rate of broilers (Fig. 2A). Besides, HS increased (*P* < 0.05) the protein expression of heat shock protein 70 (HSP70) in breast muscle of broilers (Fig. 2C). Se supplementation exhibited protective effects. Consistent with the other three Se sources, NanoSe recovered (*P* < 0.05) the protein expression of HSP70, while exhibited limited impact on the breast muscle rate of broilers.

Further test showed that HS decreased (*P* < 0.05) the muscular Se concentration (Fig. 2B) and caused poor meat quality of breast muscle, which decreased (*P* < 0.05) the pH value in 45 min and 24 h and decreased the L* value in 45 min (Fig. 2D and E), while increased (*P* < 0.05) the drip loss, cooking loss and peak shear force (Fig. 2H–J). Se supplementation increased the muscular Se concentration. However, consistent with the SS, the Se deposition efficiency of NanoSe in breast muscle was lower than that of SeY and SeMet, the deposition efficiency of these Se was SeMet > SeY > SS ≈ NanoSe (Fig. 2B). Besides, the protective effect of NanoSe on meat quality is worse than that of other Se sources, which exhibited limited improvement on the breast muscle meat quality of broilers exposed to HS, except decreased the peak shear force (Fig. 2J). While, SS, SeY and SeMet resulted (*P* < 0.05) in the higher 24 h pH value (Fig. 2D), the lower (*P* < 0.05) drip loss, cooking loss and peak shear force (Fig. 2H–J), compared with the HS group.

### Muscle fiber types and glycolysis status in breast muscle

**Fig. 3:**
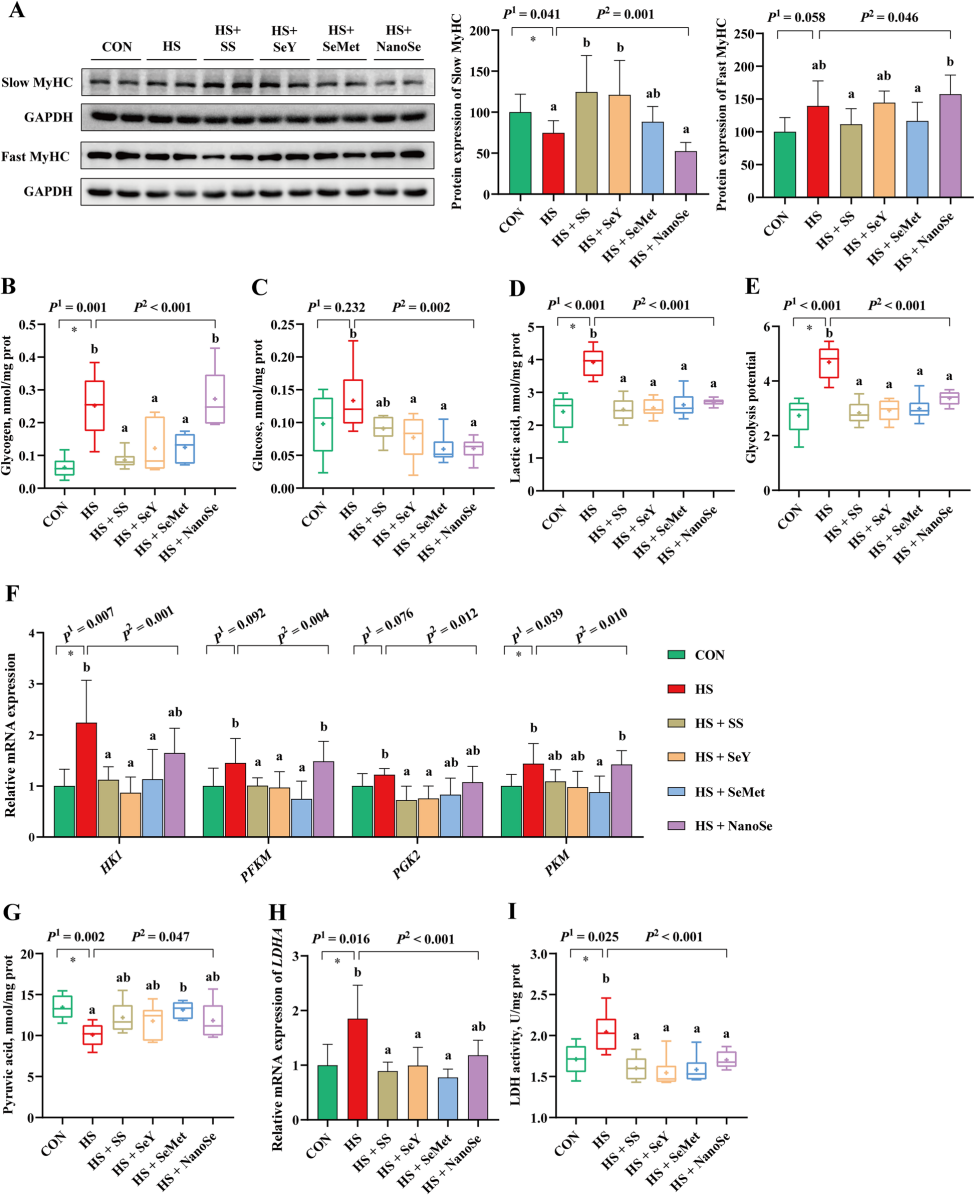
Muscle fiber types and glycolysis status of breast muscle. **A** Protein expression of Slow-MyHC and Fast-MyHC; **B** Glycogen concentration in breast muscle; **C** Glucose concentration in breast muscle; **D** Lactic acid concentration in breast muscle; **E** Glycolysis potential of breast muscle; **F** mRNA expression of *HK1*, *PFKM*, *PGK2* and *PKM*; **G** Pyruvic acid concentration in breast muscle; **H** mRNA expression of *LDHA*; **I** Activity of LDH in breast muscle. Results were expressed as means with SD or box with mean values (*n* = 6). “ + ” in the box represents the mean value. *P*^1^ was the *t*-test *P*-value between CON and HS group. *P*^2^ was the ANOVA *P*-value among these HS groups. * means there was a significantly difference between CON and HS group (*P* < 0.05). Different letters indicate significant difference between each HS group (*P* < 0.05)

The muscle fiber types and glycolysis status in breast muscle of broilers were further determined. Compared with the CON group, HS lowered (*P* < 0.05) the protein expression of Slow-MyHC, and tended to increase (*P* = 0.058) the protein expression of Fast-MyHC in breast muscle (Fig. 3A). The regulation effect of NanoSe on skeletal muscle fiber conversion was less sufficient than that of other Se sources, which exhibited no impact on the protein expression of Slow-MyHC and Fast-MyHC in breast muscle of heat stressed-broilers. While, SS, SeY and SeMet supplementation increased or tended to increase the protein expression of Slow-MyHC. SS and SeMet supplementation tended to decrease the protein expression of Fast-MyHC.

For glycolysis status, HS increased (*P* < 0.05) the concentration of glycogen, glucose and lactic acid in breast muscle (Fig. 3B–D). It is calculated that broilers exposed to HS showed the higher (*P* < 0.05) glycolytic potential (Fig. 3E). Further determination showed that HS increased (*P* < 0.05) the mRNA expression of glycolysis related-enzymes hexokinase 1 (*HK1*) and pyruvate kinase muscle type (*PKM*), and tended to increase the mRNA expression of phosphofructokinase muscle type (*PFKM*; *P* = 0.092) and phosphoglycerate kinase 2 (*PGK2*; *P* = 0.076) (Fig. 3F). Besides, the increased concentration of lactic acid in breast muscle was accompanied by the higher (*P* < 0.05) mRNA level of lactate dehydrogenase A (*LDHA*) and activity of LDH (Fig. 3H and I), while the lower (*P* < 0.05) concentration of pyruvic acid (Fig. 3G). Different Se sources supplementation showed the mitigation effects, which decreased (*P* < 0.05) the glucose, lactic acid concentration and the glycolytic potential (Fig. 3C–E), decreased the mRNA expression of *LDHA*, and suppressed (*P* < 0.05) the activity of LDH (Fig. 3I). However, compared with the other three Se sources, NanoSe group showed the higher glycogen level (Fig. 3B), and the higher mRNA levels of *HK1*, *PKM*, *PFKM* and *PGK2* in breast muscle (Fig. 3F).

### Mitochondrial tricarboxylic acid cycle in breast muscle

**Fig. 4:**
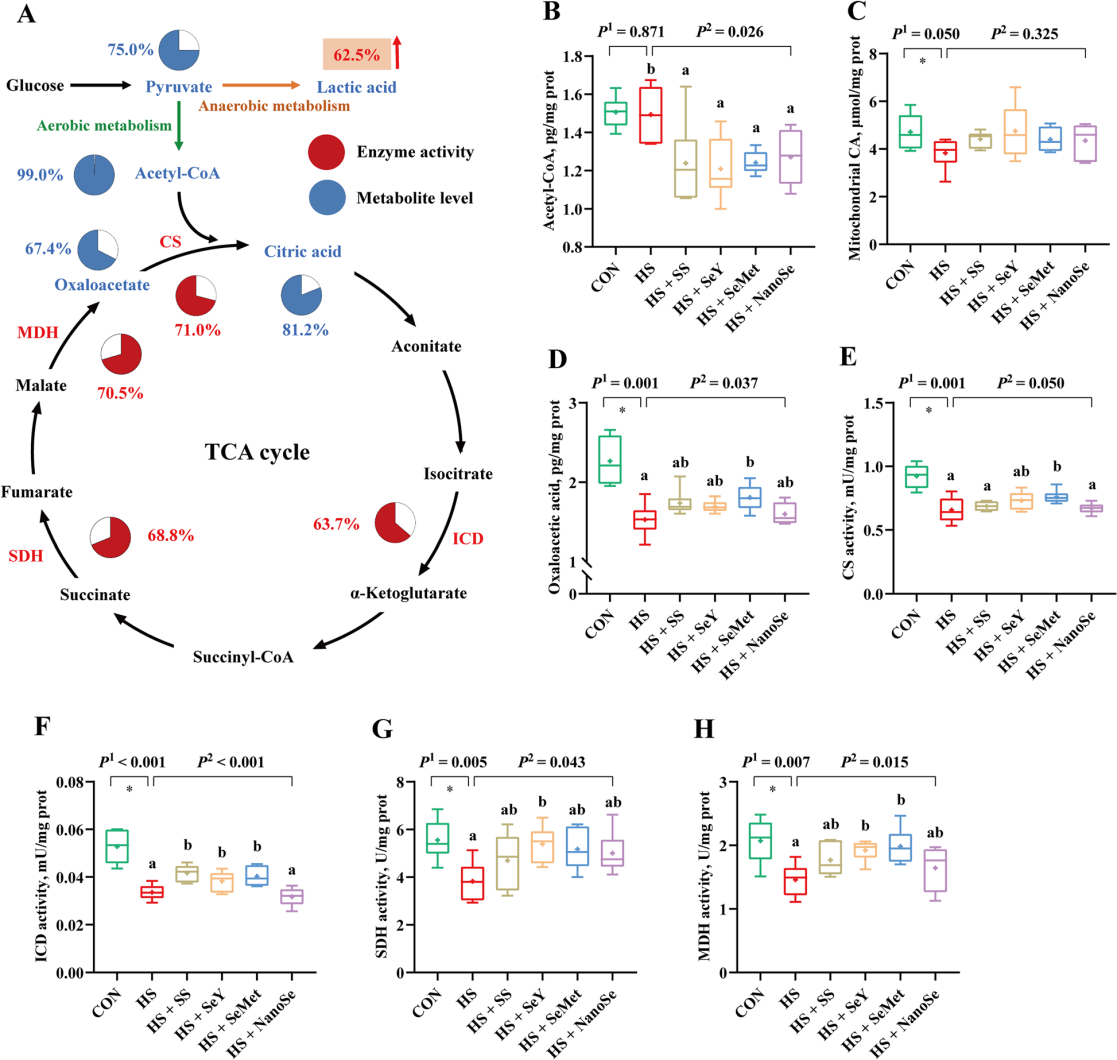
Mitochondrial tricarboxylic acid cycle status of breast muscle. **A** Changes of TCA cycle related metabolites and enzyme activities; **B** Acetyl-CoA concentration in breast muscle; **C** Mitochondrial CA concentration in breast muscle; **D** Oxaloacetic acid concentration in breast muscle; **E** Activity of CS in breast muscle; **F** Activity of ICD in breast muscle; **G** Activity of SDH in breast muscle; **H** Activity of MDH in breast muscle. Results were expressed as box with mean values (*n* = 6). “ + ” in the box represents the mean value. *P*^1^ was the *t*-test *P*-value between CON and HS group. *P*^2^ was the ANOVA *P*-value among these HS groups. * means there was a significantly difference between CON and HS group (*P* < 0.05). Different letters indicate significant difference between each HS group (*P* < 0.05)

The TCA cycle is the main metabolic pathway to consume the glycolytic product pyruvate in muscle. In the present study, HS exhibited no impact (*P* > 0.05) on the concentration of acetyl-CoA (Fig. 4A and B), while decreased the concentration of mitochondrial CA (*P* = 0.05) and oxaloacetic acid (*P* < 0.05) by 18.8% and 33.6% (Fig. 4A, C and D), respectively. Further determination showed that the enzyme activity of CS, ICD, SDH and MDH in breast muscle of broilers exposed to HS were decreased by 29.0%, 36.3%, 31.2% and 29.5% (Fig. 4A, E–H) , respectively. Different Se sources supplementation showed the mitigation effects, which decreased (*P* < 0.05) the concentration of acetyl-CoA (Fig. 4B), increased or tended to increase the concentration of oxaloacetic acid (Fig. 4D) and the activities of SDH and MDH (Fig. 4G and H). However, compared with the other three Se sources, broilers received NanoSe showed the lower activity of ICD in breast muscle (Fig. 4F). Compared with the SeY and SeMet, NanoSe supplementation showed the lower activities of CS and MDH in breast muscle of heat stressed-broilers (Fig. 4E and H).

### Antioxidant capacity and mitochondrial homeostasis of breast muscle

**Fig. 5:**
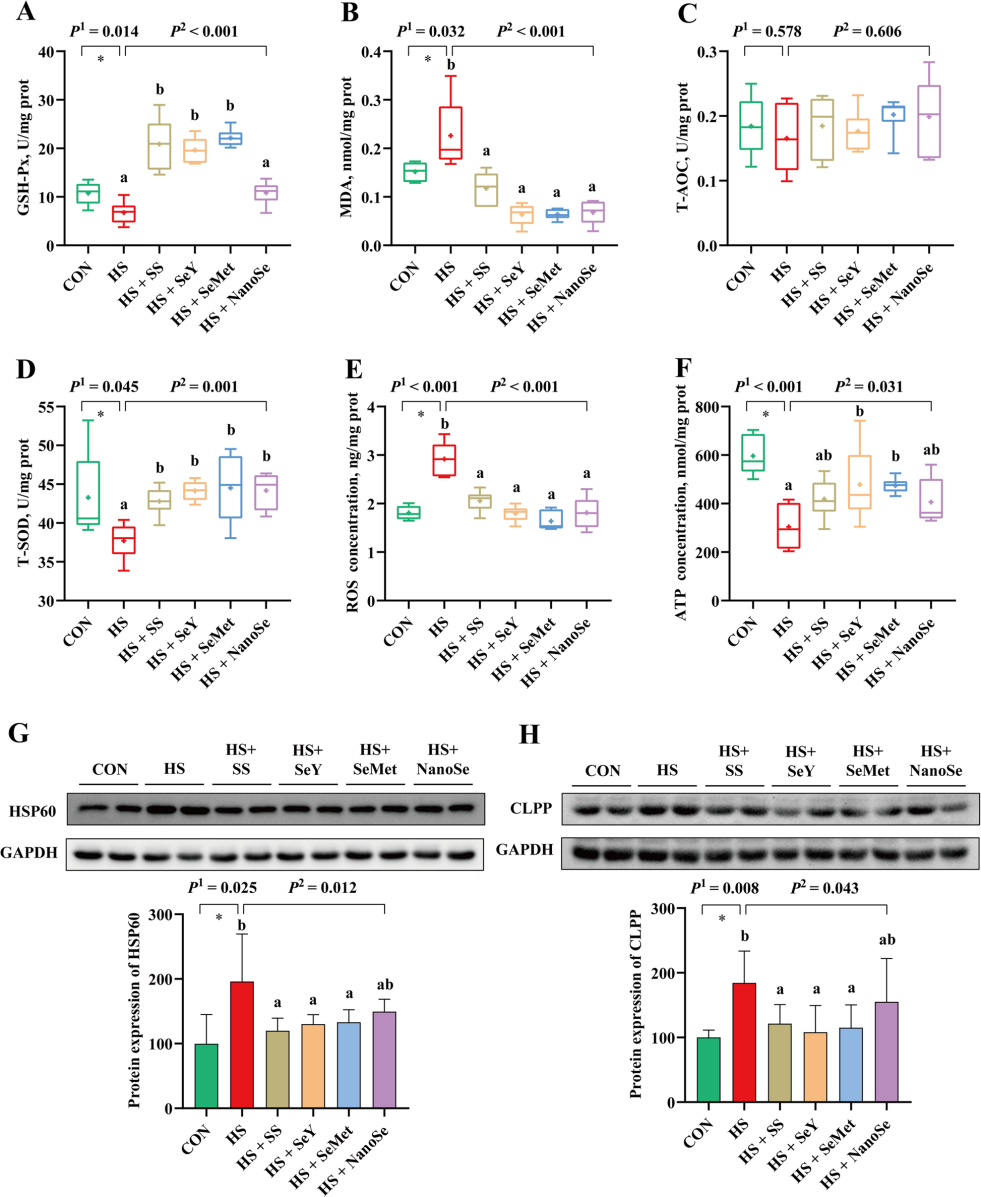
Antioxidant status and mitochondrial homeostasis of breast muscle. **A** Activity of GSH-Px in breast muscle; **B** MDA concentration in breast muscle; **C** Activity of T-AOC in breast muscle; **D** Activity of T-SOD in breast muscle; **E** ROS concentration in breast muscle; **F** ATP concentration in breast muscle; **G** Protein expression of HSP60 in breast muscle; **H** Protein expression of CLPP in breast muscle. Results were expressed as means with SD or box with mean values (*n* = 6). “ + ” in the box represents the mean value. *P*^1^ was the *t*-test *P*-value between CON and HS group. *P*^2^ was the ANOVA *P*-value among these HS groups. * means there was a significantly difference between CON and HS group (*P* < 0.05). Different letters indicate significant difference between each HS group (*P* < 0.05)

We evaluated whether HS suppressed the antioxidant capacity and caused mitochondrial stress in breast muscle, and investigated the protective effects of different Se sources. In the present study, HS reduced the antioxidant capacity of breast muscle, which decreased (*P* < 0.05) the activity of GSH-Px and T-SOD (Fig. 5A and D), while increased (*P* < 0.05) the concentration of MDA and ROS (Fig. 5B and E). Besides, HS reduced (*P* < 0.05) the concentration of ATP in breast muscle of broilers (Fig. 5F). Further determination showed that HS increased (*P* < 0.05) the protein expression of HSP60 and CLPP in breast muscle (Fig. 5G and H). Different Se sources supplementation showed the protective effects, which decreased or tended to decrease the concentration of MDA and ROS (Fig. 5B and E), and the protein expression of HSP60 and CLPP (Fig. 5G and H), while, increased or tended to increase the activity of T-SOD and the ATP concentration (Fig. 5D and F). However, compared with the other three Se sources, broilers received NanoSe showed the lowest activity of GSH-Px (Fig. 5A), and the highest levels of HSP60 and CLPP (Fig. 5G and H) in breast muscle.

### Correlation analysis

**Fig. 6:**
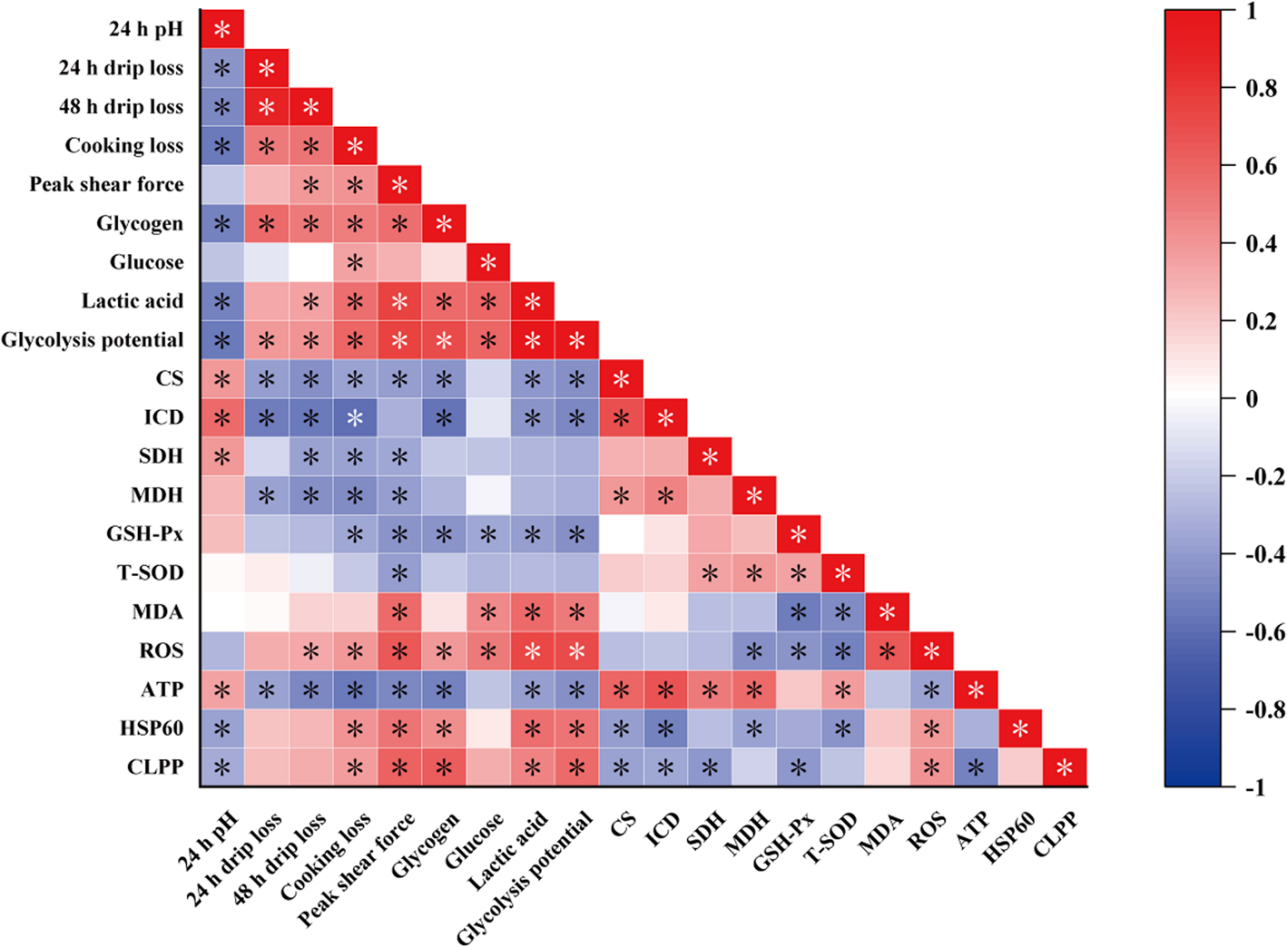
Heat map of correlations analysis. * indicates that there was a significant correlation (*P* < 0.05). The color with red represents a positive correlation, and the color with blue represents a negative correlation

We performed the correlation analysis to validate the relationship between the meat quality and glycometabolism and mitochondrial homeostasis, thus identify the reasons for the differences in the protective effects of Se sources on meat quality of broilers exposed to HS (Fig. 6). For pH value of breast muscle, the levels of glycogen, lactic acid, glycolysis potential, HSP60 and CLPP were negatively (*P* < 0.05) correlated with the pH value, while, CS, ICD, SDH and ATP were positively (*P* < 0.05) correlated with the pH value. For drip loss, the levels of glycogen and glycolysis potential were positively (*P* < 0.05) correlated with the drip loss, while, CS, ICD, MDH and ATP were negatively (*P* < 0.05) correlated with the drip loss. For cooking loss, the levels of glycogen, glucose, lactic acid, glycolysis potential, ROS, HSP60 and CLPP were positively (*P* < 0.05) correlated with the cooking loss, while, the CS, ICD, SDH, MDH, GSH-Px and ATP were negatively (*P* < 0.05) correlated with the cooking loss. For peak shear force, the levels of glycogen, lactic acid, glycolysis potential, MDA, ROS, HSP60 and CLPP were positively (*P* < 0.05) correlated with the peak shear force, while, the CS, SDH, MDH, GSH-Px, T-SOD and ATP were negatively (*P* < 0.05) correlated with the peak shear force.

### Expression of the selenotranscriptome and key selenoproteins in breast muscle

**Fig. 7:**
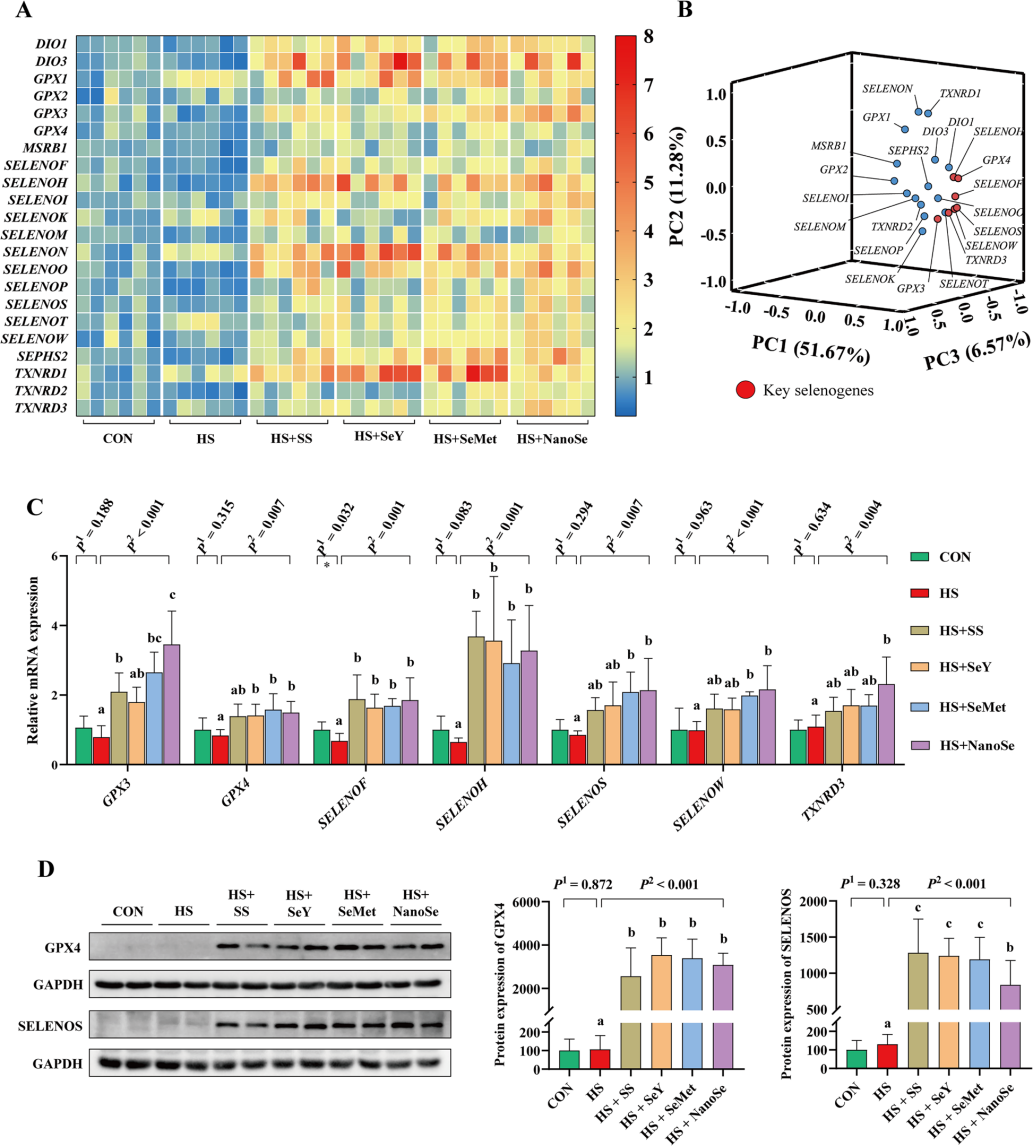
Expression of the selenotranscriptome and key selenoproteins in breast muscle. **A** Heatmap of the mRNA expression of the selenotranscriptome; **B** Principal component analysis of the selenotranscriptome; **C** Relative mRNA expression of the key selenoproteins; **D** Protein expression of GPX4 and SELENOS. Results were expressed as means with SD (*n* = 6). *P*^1^ was the *t*-test *P*-value between CON and HS group. *P*^2^ was the ANOVA *P*-value among these HS groups. * means there was a significantly difference between CON and HS group (*P* < 0.05). Different letters indicate significant difference between each HS group (*P* < 0.05)

Selenium performs its biological function mainly through selenoproteins. Here, we determined the mRNA expression of the selenotranscriptome and protein expression of several selenoproteins in breast muscle. Results showed that different Se sources supplementation all increased (*P* < 0.05) the mRNA expression of the selenotranscriptome (Fig. 7A). The principal component analysis was further performed to distinguish the key selenogenes that play main role in anti-heat stress progress. Results showed that *GPX3*, *GPX4*, *SELENOF*, *SELENOH*, *SELENOS*, *SELENOW* and *TXNRD3* were observed at the relatively distant positions (Fig. 7B), therefore, these 7 genes are key selenogenes. HS showed limited impact on the mRNA expression of these key selenogenes, except decreased (*P* < 0.05) the expression of *SELENOF* (Fig. 7C). However, different Se sources supplementation all increased (*P* < 0.05) the mRNA expression of these key selenogenes. We further chose 2 key selenogenes (*GPX4* and *SELENOS*) to verify the protein expression of them. Different Se sources supplementation all increased the protein expression of GPX4 and SELENOS (Fig. 7D). However, for the GPX4, the expression level was SeY ≈ SeMet > NanoSe > SS, for the SELENOS, the expression level was SS ≈ SeY ≈ SeMet > NanoSe.

## Discussion

In this study, we developed the heat stressed-broiler model and evaluated the protective effects of NanoSe on growth performance and meat quality of broilers exposed to HS, and compared whether there were differences between NanoSe and other Se sources. In the present study, after exposed to HS for 21 d, the final BW, ADFI and ADG of broilers exposed to HS were decreased by 26.0%, 25.1% and 36.8%, respectively. While, the F/G was increased by 19.0%. Evidences show that different Se sources or levels exhibit limited impact on the growth performance of broilers under normal conditions [21, 27]. In the present study, consistent with SS, SeY and SeMet, NanoSe supplementation at the level of 0.3 mg Se/kg showed no impact on the growth performance of broilers exposed to HS.

The breast muscle is the main edible part of the broiler. Growth restriction of broilers exposed to HS is generally accompanied by the decreased breast muscle rate [4]. Similarly, in this study, HS decreased the breast muscle rate of broilers. The HSP70 is considered as a cellular thermometer and generally used to assess HS injury [28]. In the present study, HS increased the protein expression of HSP70 in breast muscle. Which suggests that HS causes breast muscle injury and suppressed skeletal muscle development. Selenium is essential for skeletal muscle growth, Se deficiency induces nutritional muscular dystrophy of broilers [29]. Additional Se supplementation improves skeletal muscle development under oxidative stress conditions [13]. In this study, consistent with the SS, SeY and SeMet, NanoSe decreased the protein expression of HSP70 in breast muscle, and numerically increased the breast muscle rate of broilers exposed to HS. Which indicates that NanoSe may protect the breast muscle from HS damage.

Heat stress causes poor meat quality of broilers, especially lowers the pH and increases the drip loss and peak shear force [3, 30]. Here, we investigated the impact of HS on meat quality of broilers, and the protective effects of NanoSe. In the present study, HS decreased the pH value and decreased the 45 min L* value, while increased the drip loss, cooking loss and peak shear force of breast muscle. Dietary Se supplementation is an effective way to produce Se-rich livestock products, and improve meat quality of broilers [31]. In the present study, NanoSe supplementation increased the Se concentration in breast muscle. However, consistent with the SS, the deposition efficiency of NanoSe is lower than that of SeY and SeMet. Compared with SS, SeY and SeMet, the protective effect of NanoSe on breast muscle quality of broilers exposed to HS was not ideal. Though NanoSe reduced the peak shear force, it showed no improvement on pH, meat color, drip loss and cooking loss of breast muscle in broilers exposed to HS.

To explore the reasons for the unsatisfactory protective effects of NanoSe on meat quality of broilers exposed to HS, we determined the expression of muscle fiber types in breast muscle. Breast muscle with the higher concentration of fast MyHC is generally accompanied by the lower pH and higher drip loss [32]. While, muscle with the higher levels of slow MyHC generally shows the lower drip loss and higher pH value [33]. In the present study, HS decreased the protein expression of slow MyHC and increased the protein expression of fast MyHC in breast muscle. Dietary NanoSe supplementation showed limited protective effects on muscle fiber type conversion in breast muscle of broilers exposed to HS. Broilers received NanoSe showed the lower slow MyHC content and the higher fast MyHC content in breast muscle, compared with the other three Se sources. Muscle with higher fast MyHC concentration is rich in glycogen and lactic acid [34]. Elevated glycogen and lactic acid lead to a decrease in the pH value and water-holding capacity of the meat sample [11]. In this study, HS increased the concentration of glycogen and lactic acid, and increased the glycolytic potential of breast muscle in broilers. Further determination showed that HS increased the mRNA expression of glycolysis related-enzymes (*HK1* and *PKM*) and *LDHA*, and increased the enzyme activity of LDH. HK and PK encoded by *HK1* and *PKM* are key rate-limiting enzymes in the glycolysis process, HK catalyzes the first step of glucose metabolism, PK catalyzes the production of pyruvate [35, 36]. LDH rapidly converts pyruvate to lactic acid in breast muscle of birds [37]. The above results suggest that HS impairs meat quality through regulating the conversion of muscle fiber types and promoting the glycolysis. Consistent with the other three Se sources, NanoSe supplementation decreased the levels of glucose, lactic acid and glycolysis potential. However, compared with other Se sources, NanoSe showed limited impact on the concentration of glycogen and the mRNA expression of glycolysis related-enzymes in breast muscle of broilers exposed to HS. The above results revealed the potential cause that the breast muscle meat quality of broilers in the NanoSe group was not well improved compared with other Se resources.

Pyruvate decomposition mainly through two ways, producing lactic acid through anaerobic metabolism or entering the mitochondria for aerobic metabolism [38]. Previous study shows that HS induces mitochondrial stress, thus impairs the mitochondrial TCA cycle and suppresses the aerobic metabolism of pyruvate in broilers [10]. In the present study, HS decreased the concentration of pyruvate, while exhibited no impact on the concentration of acetyl-CoA in breast muscle of broilers. Besides, the concentration of lactic acid in breast muscle was increased by 62.5%, which suggest that HS promotes the anaerobic metabolism of pyruvate in breast muscle. The TCA cycle is the ultimate pathway for the aerobic metabolism of pyruvate, which is mediated by a series of enzymes, such as CS, ICD, SDH and MDH [39]. In the present study, HS decreased the activities of CS, ICD, SDH and MDH, as well as the concentration of metabolic intermediates (CA and oxaloacetic acid) of the TCA cycle. The above results suggest that HS inhibits aerobic metabolism of pyruvate through suppressing the TCA cycle, thus causes high levels of glycogen and lactic acid in breast muscle. Compared with SeY and SeMet, NanoSe supplementation showed the weak protective effects on mitochondrial TCA cycle in breast muscle of broilers exposed to HS, which showed the lowest activity of CS, ICD and MDH. Inability to effectively protect the mitochondrial TCA cycle in breast muscle under HS is a potential reason that NanoSe cannot effectively improve the breast muscle meat quality.

The proper function of the mitochondrial TCA cycle depends on the maintenance of mitochondrial homeostasis [40]. HS impairs mitochondrial homeostasis is generally accompanied by the decreased antioxidant capacity and increased ROS content [10, 41]. The impaired antioxidant capacity of muscle is generally accompanied by poor meat quality [11]. In this study, HS impaired the mitochondrial homeostasis and decreased the antioxidant capacity of breast muscle, which increased the concentration of MDA and ROS, decreased the activities of GSH-Px and T-SOD, decreased the concentration of ATP, and increased the protein expression of HSP60 and CLPP. ROS and ATP are generated from mitochondrial oxidative phosphorylation, while mitochondrial dysfunction increases the production of ROS and suppresses the synthesis of ATP [42]. HSP60 and CLPP are representative molecular chaperone and protease in the mitochondrion, which mainly involve in the maintenance of mitochondrial homeostasis and considered as the mitochondrial stress biomarkers [43, 44]. Se supplementation contributes to maintaining the mitochondrial homeostasis and enhancing the antioxidant capacity of skeletal muscle [13]. Here, we determined the effect of NanoSe on the antioxidant capacity and the expression of mitochondrial stress biomarkers in breast muscle. Consistent with the other three Se sources, NanoSe improved the antioxidant capacity of the breast muscle, which decreased the levels of MDA and ROS, and increased the activity of T-SOD. However, unlike with the other Se sources, NanoSe showed limited impact on the activity of GSH-Px, and broilers received NanoSe showed the higher levels of HSP60 and CLPP in breast muscle. The above results suggest that the regulation effect of NanoSe on mitochondrial homeostasis is less effective than that of other Se sources.

To explore the reason that NanoSe exhibited poor protective effect on meat quality of broilers exposed to HS, the correlation analysis was performed. Interestingly, the indicators (such as glycogen, CS, ICD, GSH-Px, HSP60 and CLPP) that were not effectively regulated by NanoSe were significantly correlated with the meat quality. The levels of glycogen, HSP60 and CLPP were negatively correlated with the pH, and positively correlated with the drip loss, cooking loss and peak shear force. The levels of CS, ICD and GSH-Px were positively correlated with the pH, while negatively correlated with the drip loss, cooking loss and peak shear force. The above results suggest that NanoSe cannot effectively regulate mitochondrial homeostasis, mitochondrial TCA cycle and glucose metabolism, which may be the reason that it cannot effectively protect the breast muscle meat quality of broilers exposed to HS when compared with other Se sources.

Selenium performs its biological function mainly through selenoproteins. The increased expression of selenoproteins in skeletal muscle of broilers is generally accompanied by improved meat quality [27]. Besides, the increased expression of selenoproteins contributes to maintaining the mitochondrial homeostasis in liver of broilers or skeletal muscle of pigs [10, 13]. Generally, the expression of selenoproteins in muscle of animals is effectively regulated by the diet Se concentration [16, 17]. Here, we explored the mRNA expression of the selenotranscriptome in breast muscle of broilers that received NanoSe and other Se sources. Consistent with other three Se sources, NanoSe supplementation increased the mRNA expression of the selenotranscriptome. For the screened key selenoproteins, NanoSe also increased the mRNA expression of these selenoproteins. For these key selenogenes, we selected GPX4 and SELENOS to investigate their protein expression. GPX4 plays an important role in the regulation of cell homeostasis, and mitochondrial GPX4 (mGPX4) primarily resides in mitochondria and contributes to the maintenance of mitochondrial homeostasis [45]. Increased expression of GPX4 is accompanied by improved antioxidant capacity and meat quality [16]. SELENOS contribute to the maintenance of cell homeostasis by participating in protein quality control, and increased expression of SELENOS contributes to improving the breast muscle meat quality of heat stressed-broilers [16, 46]. In the present study, NanoSe supplementation increased the protein expression of GPX4 and SELENOS. However, compared with the other three Se sources, broilers received NanoSe showed the lowest protein expression of SELENOS. Besides, consistent with the Se deposition efficiency, the expressions of GPX4 in breast muscle of broilers received SS and NanoSe were numerically lower than that of SeY and SeMet. The above results suggest that NanoSe could not effectively increase the protein expression of key selenoproteins such as SELENOS, compared with other Se sources.

## Conclusion

The present study reveals that HS impairs mitochondrial homeostasis, lowers the antioxidant capacity, suppresses the mitochondrial TCA cycle, promotes the anaerobic metabolism of pyruvate, thus causes poor meat quality of breast muscle in broilers. Dietary different Se sources supplementation increase the Se concentration and promote the expression of several key selenoproteins in breast muscle of broilers. The increased expression of these key selenoproteins improve the mitochondrial homeostasis, increase the antioxidant capacity, promote the mitochondrial TCA cycle and the aerobic metabolism of pyruvate, thus improve the breast muscle meat quality of broilers exposed to HS. The mitigation effects of organic Se (SeY and SeMet) are better than that of SS and NanoSe. NanoSe cannot effectively regulate mitochondrial homeostasis, mitochondrial TCA cycle and glucose metabolism in breast muscle, thus shows the weak protective effect against HS induced poor breast muscle meat quality of broilers compared with the other Se sources.

### Supplementary Information

**Additional file 1: Fig. S1** Se concentration in diet. Results were expressed as mean ± SD (*n* = 4).

**Additional file 2: Table S1** Primers used for the RT-PCR. **Table S2** Primary antibodies for the western blot analysis.

## Data Availability

All data generated or analyzed during this study are included in this published article and its supplementary information files.

## Abbreviations

ADFI: Average daily feed intake
ADG: Average daily body gain
ATP: Adenosine triphosphate
BW: Body weight
CA: Citric acid
CLPP: Caseinolytic mitochondrial matrix peptidase
CON: Control group
CS: Citroyl synthetase
ELISA: Enzyme-linked immunosorbent assay
F/G: Feed intake/body gain
GSH-Px: Glutathione peroxidase
HK1: Hexokinase 1
HS: Heat stress
HSP60: Heat shock protein 60
HSP70: Heat shock protein 70
ICD: Isocitrate dehydrogenase
LDH: Lactic dehydrogenase
MDA: Malonaldehyde
MDH: Malic dehydrogenase
NanoSe: Nano selenium
OA: Oxaloacetate
PFKM: Phosphofructokinase muscle type
PGK2: Phosphoglycerate kinase 2
PKM: Pyruvate kinase muscle type
PMSF: Phenylmethanesulfonyl fluoride
RIPA: Radio immunoprecipitation assay
ROS: Reactive oxygen species
SDH: Succinodehydrogenase
Se: Selenium
SeMet: Selenomethionine
SeY: Selenoyeast
SS: Sodium selenite
T-AOC: Total antioxidant capacity
TCA: Tricarboxylic acid
T-SOD: Total superoxide dismutase
