# Supplementary figures and images for: Nano-Se exhibits limited protective effect against heat stress induced poor breast muscle meat quality of broilers compared with other selenium sources
Source: J Anim Sci Biotechnol. 2024 Jul 8;15:95. doi: 10.1186/s40104-024-01051-2 (PMC11229195; doi:10.1186/s40104-024-01051-2)

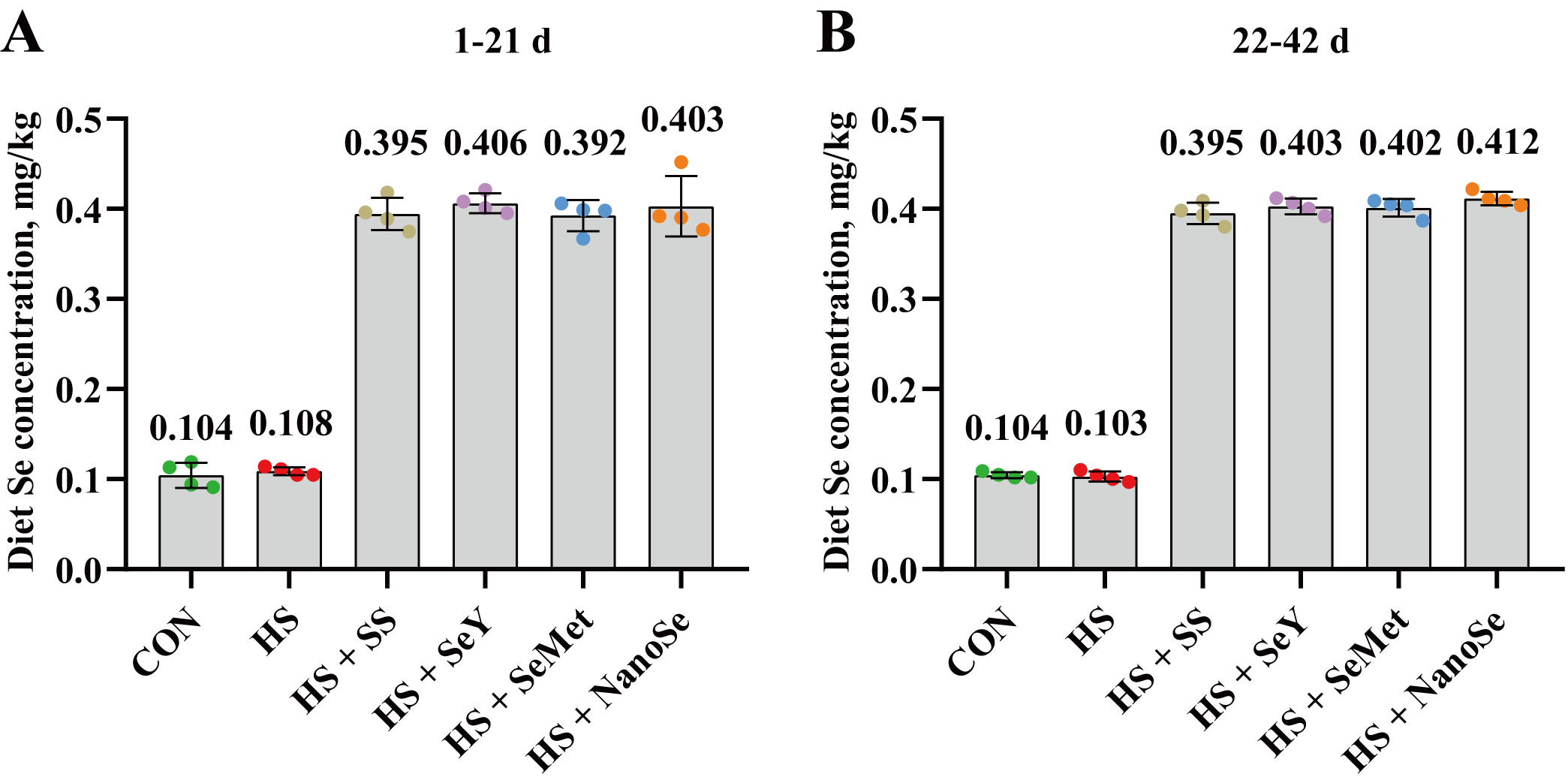

Supplement: Supplementary file 1 — Additional file 1: Fig. S1 Se concentration in diet. Results were expressed as mean ± SD (n = 4). [file 40104_2024_1051_MOESM1_ESM.tif]
