# Supplementary material for: Nano-Se exhibits limited protective effect against heat stress induced poor breast muscle meat quality of broilers compared with other selenium sources
Source: J Anim Sci Biotechnol. 2024 Jul 8;15:95. doi: 10.1186/s40104-024-01051-2 (PMC11229195; doi:10.1186/s40104-024-01051-2)
Supplement: Supplementary file 2 — Additional file 2: Table S1 Primers used for the RT-PCR. Table S2 Primary antibodies for the western blot analysis. [file 40104_2024_1051_MOESM2_ESM.docx]

**Additional Table S1 Primers used for the RT-PCR**

| Gene | NCBI Sequence | Sequence | | Product size (bp) |
| --- | --- | --- | --- | --- |
| *β-Actin* | NM_205518.2 | F: | TATTGCTGCGCTCGTTGTTG | 52 |
|  |  | R: | GAAACCGGCCTTGCACATAC |  |
| *HK1* | NM_204101.2 | F: | GCTCCTGGCCTACTACTTCAC | 195 |
|  |  | R: | GACCTCACAAAGGTAGGGAGC |  |
| *PFKM* | NM_204223.2 | F: | CCCATTTCCCTCTTTTTCAGCC | 196 |
|  |  | R: | GCCCTCATGCACGAAGTAGA |  |
| *PGK2* | NM_204985.4 | F: | CAGCGGTGACTTGCGATAAC | 94 |
|  |  | R: | AGTCAACCCTCATGACGACC |  |
| *PKM* | NM_205469.2 | F: | CAGCAGGAGACACCGAACTC | 75 |
|  |  | R: | TGCTGGGTCTGGATGAAAGC |  |
| *LDHA* | NM_205284.2 | F: | TGTCTGGAGCGGAGTGAATG | 125 |
|  |  | R: | CCTCATAGGCACTGTCCACC |  |
| *DIO1* | NM_001097614.2 | F: | TCTACAAGGGAGGAGTGGGG | 62 |
|  |  | R: | TTCCAGGACAGCGCGTATTT |  |
| *DIO2* | NM_001324555.3 | F: | TGCGCGCGGTCAAACTT | 64 |
|  |  | R: | TTGCCCTTGGCTATGTGGATT |  |
| *DIO3* | NM_001122648.3 | F: | CAGTACAAAACCCGGCTCCA | 50 |
|  |  | R: | TACACTTGGATGACCACCGC |  |
| *GPX1* | NM_001277853.3 | F: | ACCATGTTCGAGAAGTGCGA | 65 |
|  |  | R: | TCTCTCAGGAAGGCGAACAG |  |
| *GPX2* | NM_001277854.3 | F: | GGTCCTCATCGAGAACGTGG | 59 |
|  |  | R: | GCTGGGTGTAATCCCTCACC |  |
| *GPX3* | NM_001163232.3 | F: | CACCATCTACGACTACGGGG | 74 |
|  |  | R: | TCTTCCCCGCGTACTTTCTG |  |
| *GPX4* | NM_001346449.2 | F: | GTGATGCTCCCCTTCGTCTC | 56 |
|  |  | R: | TACAGGTAGGCGGGCAGAT |  |
| *MSRB1* | NM_001135558.3 | F: | CCCGCAGGAGAATTAAGCGA | 76 |
|  |  | R: | GCTCCCATACACCCTGACTG |  |
| *SELENOF* | NM_001012926.3 | F: | AGTACGTGCGTGGTTCTGAC | 58 |
|  |  | R: | GCAATGTTCCCACTGTCGTC |  |
| *SELENOH* | NM_001277865.2 | F: | GCCGTAGAGATCAACCCGC | 50 |
|  |  | R: | GACACCTCGAAGCTGTTCCT |  |
| *SELENOI* | NM_001031528.4 | F: | TGCCAGCCTCTGAACTGGAT | 69 |
|  |  | R: | TGCAAACCCAGACATCACCAT |  |
| *SELENOK* | NM_001025441.2 | F: | ATAAATCACTGGGGTGGAGGC | 93 |
|  |  | R: | GCTCCTTTGCCTGCTTCTTAC |  |
| *SELENOM* | NM_001277859.2 | F: | ACATCCCGCTGTACCATAACCT | 126 |
|  |  | R: | TCTCCTCCCGGGTCATGTC |  |
| *SELENON* | NM_001114972.3 | F: | CTGTATGGGGCGAGTGAAGG | 99 |
|  |  | R: | TATCACAGAGGGGACCGAGG |  |
| *SELENOO* | NM_001115017.5 | F: | CCCAGCGTTAACCGGAATGA | 147 |
|  |  | R: | TCTTGCCGTCCGCTTTGTTA |  |
| *SELENOP* | NM_001031609.3 | F: | CCAAGTGGTCAGCATTCACATC | 81 |
|  |  | R: | ATGACGACCACCCTCACGAT |  |
| *SELENOS* | NM_001024734.3 | F: | CCGACATGGTGGTAAGAAGACA | 76 |
|  |  | R: | GCTTGTGCATTCAACTCCTCTTG |  |
| *SELENOT* | NM_001006557.4 | F: | GATCTGCGTCTCCTGAGGTT | 97 |
|  |  | R: | GTAGTTCTCCCCCTCGATGC |  |
| *SELENOU* | NM_001193519.3 | F: | GGCTGCTTCGGAAATGTCT | 95 |
|  |  | R: | CTGTTATGGCTGCGCCAAC |  |
| *SELENOW* | NM_001166327.2 | F: | TGTGGGTCTGCTTTACGCC | 70 |
|  |  | R: | AAGCTGGAAGGTGCAAAATGAA |  |
| *SEPHS2* | NM_001366334.2 | F: | AGGTTGACGAGTTACAGCGA | 51 |
|  |  | R: | CTCCGGCACTTTACAGCCTC |  |
| *TXNRD1* | NM_001030762.4 | F: | GCCTTACTGTCCGGGGAAAA | 63 |
|  |  | R: | CCTGCACATTCCAAGGCAAC |  |
| *TXNRD2* | NM_001122691.3 | F: | AAAGATGCCCAGCACTACGG | 71 |
|  |  | R: | GCTTGAGCCATCACAGACCA |  |
| *TXNRD3* | NM_001122777.3 | F: | TGACCTCTTCTCCCTGCCTTA | 53 |
|  |  | R: | AAGCACCCACAACTAGCGTT |  |

**Additional Table S2 Primary antibodies for the** **western blot analyses**

| Antibody | Company | Address | Commodity code | Dilution ratio |
| --- | --- | --- | --- | --- |
| HSP70 | Proteintech Group | Illinois, USA | 10995-1-AP | 1:5000 |
| Slow MyHC | Servicebio | Wuhan, China | GB111857-100 | 1:1000 |
| Fast MyHC | Servicebio | Wuhan, China | GB112130-100 | 1:1000 |
| CLPP | Zen BioScience | Chengdu, China | R23947 | 1:1000 |
| GPX4 | Zen BioScience | Chengdu, China | 513309 | 1:2000 |
| HSP60 | Proteintech Group | Illinois, USA | 66041-1-Ig | 1:5000 |
| SELENOS | Proteintech Group | Illinois, USA | 15591-1-AP | 1:1000 |
| GAPDH | Proteintech Group | Illinois, USA | 60004-1-Ig | 1:10000 |
